# Gene Expression Profiling of Gastric Cancer

**Published:** 2011-04-16

**Authors:** Arivusudar Marimuthu, Harrys K.C. Jacob, Aniruddha Jakharia, Yashwanth Subbannayya, Shivakumar Keerthikumar, Manoj Kumar Kashyap, Renu Goel, Lavanya Balakrishnan, Sutopa Dwivedi, Swapnali Pathare, Jyoti Bajpai Dikshit, Jagadeesha Maharudraiah, Sujay Singh, Ghantasala S Sameer Kumar, M. Vijayakumar, Kariyanakatte Veeraiah Veerendra Kumar, Chennagiri Shrinivasamurthy Premalatha, Pramila Tata, Ramesh Hariharan, Juan Carlos Roa, T.S.K Prasad, Raghothama Chaerkady, Rekha Vijay Kumar, Akhilesh Pandey

**Affiliations:** 1 Institute of Bioinformatics, International Technology Park, Bangalore, 560066, India; 2 Manipal University, Madhav Nagar, Manipal, Karnataka 576104; India; 3 McKusick-Nathans Institute of Genetic Medicine, Johns Hopkins University School of Medicine, Baltimore 21205, Maryland, USA; 4 Department of Biological Chemistry, Johns Hopkins University School of Medicine, Baltimore 21205, Maryland, USA; 5 Department of Zoology, Gauhati University, Guwahati 781014, Assam, India; 6 Imgenex India, Bhubaneswar 751024, Orissa, India; 7 Rajiv Gandhi University of Health Sciences, Bangalore, 560041, Karnataka, India; 8 Department of Biotechnology, Kuvempu University, Shimoga 577451, Karnataka, India; 9 School of Biotechnology, Amrita Vishwa Vidyapeetham University, Kollam 690525, Kerala, India; 10 Strand Life Sciences, Bangalore 560024, Karnataka, India; 11 RajaRajeswari Medical college, Bangalore, 560074, India; 12 Imgenex Corporation, San Diego 92121, California, USA; 13 Departments of Surgical Oncology, Kidwai Memorial Institute of Oncology, Bangalore 560029, Karnataka; India; 14 Department of Pathology, Kidwai Memorial Institute of Oncology, Bangalore 560029, Karnataka; India; 15 Department of Pathology, Universidad de La Frontera, Temuco, Chile; 16 Department of Oncology, Johns Hopkins University School of Medicine, Baltimore 21205, Maryland, USA; 17 Department of Pathology, Johns Hopkins University School of Medicine, Baltimore 21205, Maryland, USA

**Keywords:** DNA microarray, GeneSpring GX, Gastric cancer, Immunohistochemistry

## Abstract

Gastric cancer is the second leading cause of cancer death worldwide, both in men and women. A genomewide gene expression analysis was carried out to identify differentially expressed genes in gastric adenocarcinoma tissues as compared to adjacent normal tissues. We used Agilent’s whole human genome oligonucleotide microarray platform representing ~41,000 genes to carry out gene expression analysis. Two-color microarray analysis was employed to directly compare the expression of genes between tumor and normal tissues. Through this approach, we identified several previously known candidate genes along with a number of novel candidate genes in gastric cancer. Testican-1 (*SPOCK1*) was one of the novel molecules that was 10-fold upregulated in tumors. Using tissue microarrays, we validated the expression of testican-1 by immunohistochemical staining. It was overexpressed in 56% (160/282) of the cases tested. Pathway analysis led to the identification of several networks in which *SPOCK1* was among the topmost networks of interacting genes. By gene enrichment analysis, we identified several genes involved in cell adhesion and cell proliferation to be significantly upregulated while those corresponding to metabolic pathways were significantly downregulated. The differentially expressed genes identified in this study are candidate biomarkers for gastric adenoacarcinoma.

## Introduction

Gastric adenocarcinoma arises from the glandular epithelium (mucosa) of the stomach. More than 90 percent of gastric cancers have been reported to be adenocarcinomas with the remainder being non-Hodgkin’s lymphomas, leiomyosarcomas, gastrointestinal stromal tumors and carcinoid tumors [1]. Histologically, gastric adenocarcinoma can be classified into two categories. The first is the intestinal type characterized by cohesive neoplastic cells forming gland like tubular structures while the second type is diffuse with a thickening of the stomach wall without a discrete mass [2].

Multiple factors contribute to the progression of gastric tumors. *H. pylori* infection is one of the best known risk factors associated with gastric adenocarcinoma [3], Diet also plays a major role as a risk factor in gastric carcinogenesis. The dietary risk factors include consumption of salted, smoked or poorly preserved foods, low consumption of fruits and vegetables. Other factors associated with an increased risk of gastric cancer include chronic atrophic gastritis, hypertrophic gastropathy (Menetrier’s disease), gastric polyps, low socioeconomic status, obesity, and blood type A [4]. According to global cancer statistics, gastric cancer is the fourth most frequent type of neoplasm and second most important cause of death due to cancer [5]. Five year survival rate for gastric cancer has been reported be less than 7% [6]. Patients with gastric cancer are often diagnosed at an advanced stage since the development of tumor is often asymptomatic.

Over the past decade, a number of molecular studies have been carried out in cancers to understand disease progression and to discover biomarkers for diagnosis and prognosis. Gene expression profiling of gastric cancers has been performed by several groups using cDNA [7-11] and oligonucleotide microarray platforms [12-16]. These high-throughput studies have led to the identification of a few markers that are associated with specific histological subtypes of gastric cancer. For instance, E-cadherin, EGFR, VEGF and alpha, beta and gamma catenins have been found to distinguish the diffuse from intestinal type of gastric cancer [17]. Aberrant expression of EGFR or VEGF and amplification of *HER2* or c-MET have been described to be useful for clinical prognosis of gastric cancer [17]. Though many studies have been carried out at the molecular level on gastric cancer, it still remains poorly understood. Due to the lack of specific therapeutic targets, cytotoxic therapy remains the standard mode of treatment for unresectable gastric cancer patients and as adjuvant treatment for operable cases. This emphasizes the need for more studies at the molecular level to discover suitable biomarkers for diagnosis, prognosis and therapy.

In this study, we carried out gene expression analysis of gastric adenocarcinoma along with adjacent normal tissues. We found many genes that were differentially expressed. We validated two markers, VIL1 and SPOCK1, by immunohistochemical analysis using tissue microarrays. VIL1 was overexpressed in 76% (217/282) while SPOCK1 was overexpressed in 56% (160/282) of the tested cases, respectively.

## Materials and Methods

### Tissue samples

Surgically resected gastric adenocarcinoma samples and their paired adjacent disease-free non-malignant tissues were collected from 14 patients after obtaining Institutional Review Board (IRB) approval from the Kidwai Memorial Institute of Oncology, Bangalore. The patients who were operated on were all previously untreated (i.e. no chemotherapy or radiotherapy) with a resectable primary gastric tumor. The adjacent normal mucosa harvested at least 5 cm away from the tumor served as a normal control from the same individual [18,19]. The mucosa was sampled carefully by an expert pathologist to avoid the muscular/perimuscular tissue content at the surgical margin. The samples were immediately stored in RNA*later* (Ambion Inc., Austin, TX) and incubated overnight at 4°C to allow proper penetration of RNA*later* into the tissues after which they were transferred to -80°C.

### RNA isolation

RNA was isolated using the RNAeasy Kit (Qiagen,Valencia, CA) from 15 mg of tissue. The tissue was pulverized by grinding with liquid Nitrogen in a frozen mortar and pestle ensuring that the tissue did not thaw until it was placed in the RLT lysis buffer supplied with the kit (Buffer RLT and β-mercaptoethanol). The powdered tissue was allowed to thaw and transferred to nuclease free tubes and processed essentially as previously described [20]. Briefly, the quality of total RNA and its integrity was assessed using the Bioanalyzer 2100 (Agilent, Palo Alto, CA) and RIN value (RNA Integrity Number) was recorded for all the samples for intact 18S and 28S rRNA. Total RNA (800 ng) from each sample was reverse transcribed and linear amplification carried out using the low RNA input linear amplification kit (Agilent Technologies). After synthesis of the first and second strands of cDNA, the product was used in an *in vitro* transcription reaction to generate cRNAs in the presence of cyanine 3 (Cy3) in the case of normal or cyanine 5 (Cy5) for tumor labeled UTP (Perkin Elmer). The labeled cRNA was purified using RNeasy spin columns (Qiagen, Valencia, CA) to remove excess free nucleotides. All samples with specific activity >11.0 were considered suitable for hybridization.

### Hybridization, scanning and data analysis

Fragmented Cy3-labeled cRNA of the control sample was mixed with equal amounts of Cy5-labeled cRNA from the gastric tumor sample and the mixtures hybridized onto 44K whole human genome DNA microarrays (G4112F, 4x44K, Agilent Technologies) for 17 hrs at 65°C with constant rotation (10 rpm). Subsequently, the arrays were washed according to manufacturer’s instructions. The slides were scanned using an Agilent microarray scanner (G2505B), and the images processed and analyzed using the Agilent feature extraction software AFE 9.5. GeneSpring GX v11.0.2 (Agilent technologies) was used to analyze the expression profiles obtained after microarray hybridization. Following Lowess normalization, the data was subjected to statistical analysis. T test was performed to determine significance of the differences observed between the normal and tumor samples. This was further subjected to Benjamini Hochberg multiple testing correction to compute false discovery rates. Genes were filtered based on p-value threshold of 0.001 and false discovery rate of less than 1%. The obtained list of genes was further filtered by subjecting it to fold-change ≥2.0.

### Data submission

The raw data and the processed data from this study have been deposited into the Gene Expression Omnibus (GEO) public repository (accession number - GSE22804).

### Gene enrichment analyses

Gene Set Enrichment Analysis (GSEA) [21] was performed using GSEA-preranked method on list of differentially expressed genes. A search was done on C1, C2, C3, C4 and C2-canonical pathway gene sets available from molecular signatures database (MsigDB). Default parameters as described by Subramanian et al. [21] were used. Enriched gene sets with false discovery rate (FDR) less than 10% were selected for further evaluation.

### Biological network analysis

Pathway analysis was carried out using Genespring GX v.11.0.2. Differentially expressed genes obtained after filtering based on fold-change cut off (FC>=2.0) were taken as the input list. Biological networks were generated by comparing the input list to the reference list, which contains more than 1.4 million reactions derived from natural language processing-based extraction from literature and from different interaction databases. High confidence networks were further generated by applying filters that included binding, expression, metabolism, transport, promoter binding and regulation category of molecules. The number of molecules per network was restricted to 50. The entities which did not have connections were removed from the network. The constructed network was overlaid on the final input list to visualize the upregulated and downregulated genes. Further, *SPOCK1* and *CLDN1* genes with their corresponding subnetworks were selected. Using expand and shortest connect algorithms; the interactive pathways between them were obtained.

### Immunohistochemical staining

Custom tissue arrays prepared by JCR (Universidad de La Frontera, Temuco, Chile) and commercially available tissue microarrays (Folio biosciences # ARY-HH0201) were used for the analysis. Immunohistochemical staining was carried out essentially as previously described [20]. Briefly, formalin fixed paraffin embedded tissue sections were deparaffinized and antigen retrieval was performed for 20 minutes in antigen retrieval buffer. Endogenous peroxidases were quenched using a blocking solution, followed by washes with wash buffer (Phosphate buffered saline with 0.05% Tween). The sections were incubated with primary antibody overnight at 4°C. Anti-villin-1, rabbit polyclonal antibody was procured from Sigma (HPA006685). Anti-testican-1, rabbit polyclonal antibody was purchased from Abcam (ab83768). Anti-villi-1 and Anti-testican-1 antibodies were used at 1:500 and 1:2000 dilution, respectively. Following incubation with respective primary antibodies, the sections were rinsed with wash buffer followed by incubation with horseradish peroxidase conjugated appropriate secondary antibody. Excess of the secondary antibody was washed with wash buffer followed by addition of DAB substrate. The signal was then developed using DAB chromogen (DAKO). Tissue sections were then observed under the microscope. The immunohistochemical labeling was assessed by an experienced expert pathologist and the intensity of staining scored as negative (0), moderate (2+) or strong (3+).

## Results and Discussion

**Figure 1:**
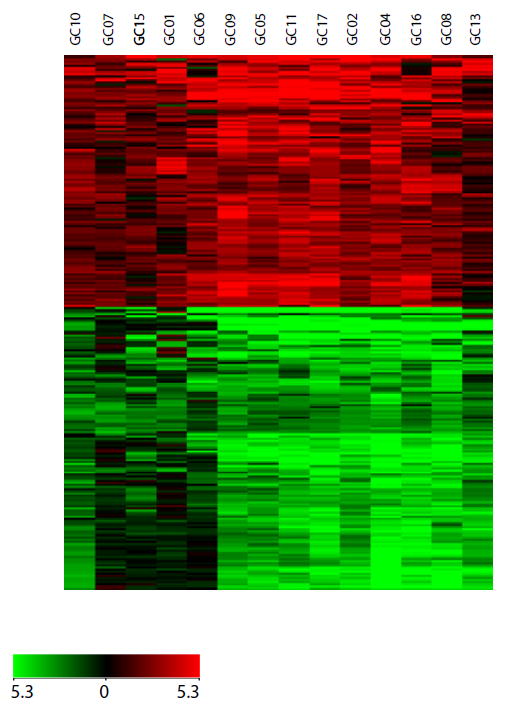
Heat map of differentially expressed genes in gastric cancer Unsupervised hierarchical clustering was performed on gene expression profiles of fourteen cases of gastric adenocarcinoma tumors and their adjacent normal. The heat map of differentially expressed genes based on clustering is shown in the figure. Each column represents a specimen and each row represents a gene. Red color indicates genes that were upregulated and green color indicates genes that were downregulated. Black indicates genes whose expression is unchanged in tumors as compared to normal.

Gene expression analysis was carried out using tumor tissues and corresponding adjacent normal tissues from gastric adenocarcinoma patients. The clinicopathological data for the patients recruited in this study are provided in Supplementary Table 1. Dual color gene expression analysis was carried out using whole human genome microarrays which were hybridized with tumor derived Cy5 labeled mRNA and normal derived Cy3 labeled mRNA. GeneSpring GX v.11.0.2 software was used for microarray data analysis. Genes were filtered based on p-value cut off of <0.001 and false discovery rate of <1%. Based on a fold-change cut off of 4, we observed 232 genes to be upregulated while 221 genes were downregulated in tumor tissues as compared to normal tissues. Next, we carried out an unsupervised hierarchical clustering of differentially expressed genes across 14 patient samples. Genes that were upregulated were clustered into one group and the genes that were downregulated clustered into another group as shown in Figure 1.

### Previously known genes upregulated in gastric cancer

Among those genes that were upregulated, we found several genes that were also reported by previous studies validating our data. A few of these genes are discussed briefly below:

Claudins belong to the family of tight junction proteins involved in maintaining cell polarity in epithelial and endothelial cells [22]. In humans, 24 claudin subtypes are known of which claudin 1-4 and 6 have been reported to be overexpressed in gastric cancer [23,24]. Claudin 1 and claudin 6 were previously reported to be overexpressed more frequently in intestinal subtype of gastric cancer as compared to the diffuse type [25,24]. In our study, claudin 1 was found to be 22-fold upregulated while claudin 4 and claudin 6 were found to be 3-fold upregulated in gastric adenocarcinoma. Osteopontin (*SPP1*) is a secreted N-linked glycoprotein known to be expressed in the epithelia of many tissues [26]. It has been shown to be overexpressed in gastric cancer [27], promote metastasis [28] and serve as a prognostic factor [26] for gastric cancer. In our study, it was found to be 14-fold upregulated. Other markers that were significantly upregulated and have been previously described in the context of gastric cancer include sulfatase 1 (*SULF1*) [29] and high mobility group AT-hook 2 (*HMGA2*) [30] which were both 8-fold upregulated, inhibin, beta A *INHBA* [31], and villin-1 (*VIL1*) [32] which were both 9-fold upregulated in gastric carcinoma tissues. A partial list of the previously reported genes is provided in Table 1 and a complete list of upregulated genes is provided in Supplementary Table 2.

### Novel genes upregulated in gastric cancer

In this study, we found a number of genes that have not been described in the context of gastric cancer and are novel. Among these, some of the genes that were upregulated are discussed in detail below

We identified a number of genes that were highly upregulated with no known previous association with any cancer. Clarin 3 (*CLRN3*), odd skipped related 2 (OSR2) and testican-1 (*SPOCK1*) are a few of the notable genes in this category. Clarin 3 is a transmembrane protein whose function is not known. Odd skipped related 2 belongs to a family of transcription factors and is a human homolog of drosophila odd skipped family of proteins [33]. *CLRN3* was found to be 12.6-fold upregulated and *OSR2* was found to be 12.9-fold upregulated in gastric adenocarcinoma as compared to normal. We also identified many genes that were known in other cancers but not reported in gastric cancer. Chordin like 2 (*CHRDL2*) is an extracellular matrix protein which is also called as breast novel factor 1. It was initially identified as a novel gene overexpressed in breast tumors by differential display analysis and later described to be overexpressed in lung and colon cancers [34]. In our study, *CHRDL2* was 7-fold upregulated in gastric cancer. Gremlin 1(*GREM1*) belongs to the family of bone morphogenic protein antagonists. It is a secreted glycoprotein known to be involved in regulation of early development [35]. It has been reported to promote tumor cell proliferation in basal cell carcinomas [36] and known to be overexpressed in carcinomas of lung, ovary, uterine cervix, colon, pancreas and sarcoma [35]. It was found to be 4.5-fold upregulated in our study. A partial list of novel genes upregulated in gastric cancer is provided in Table 2.

### Downregulated genes in gastric cancer

**Figure 2:**
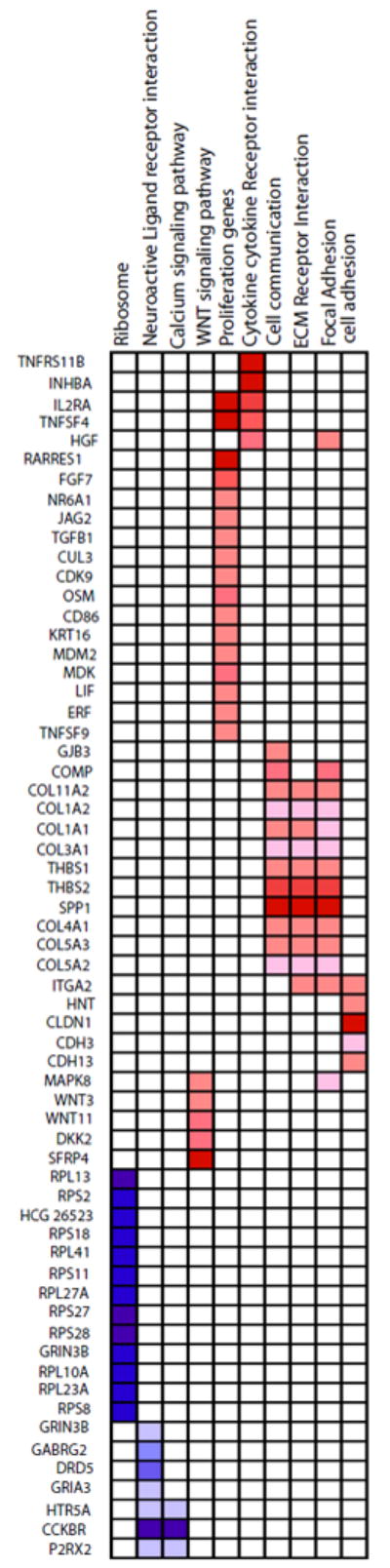
Heat map of pathways enriched in gastric cancer by GSEA analysis Pathways that were enriched by GSEA and the genes that led to their enrichment are shown. Downregulated genes are represented in shades of blue while upregulated genes are represented in shades of red.

Using a p-value threshold of 0.001 and fold-change cut off of 4, we identified 221 genes that were downregulated. A number of genes that were found to be downregulated in our study were also reported by previous studies on gastric cancer. Gastric Lipase (*LIPF*) belongs to the family of lipases which are involved in the digestion of triacylglycerides. It has been shown to be secreted specifically by the gastric mucosal cells [37]. It has been reported to be downregulated in gastric cancer and has also been reported as a part of the 8 gene signature known to predict gastric cancer [31]. In this study, *LIPF* was found to be 14-fold downregulated. Potassium voltage-gated channel, Isk-related family, member 2 (*KCNE2*) belongs to the family of voltage gated potassium ion channels which are involved in multiple cellular functions which includes neurotransmitter release, heart rate, neuronal excitability and electrolyte transport [38]. Deletion of *KCNE2* in mice has been shown to cause gastritis which is a predisposing factor for gastric cancer [39]. In another study by Yanglin et al. [40], KCNE2 was shown to be downregualted in cancer tissues and induced expression has shown to inhibit proliferation of gastric cancer [40]. In this study, we found 36-fold downregulation of *KCNE2* in gastric adenocarcinoma. Some of the novel genes that were found to be downregulated in gastric adenocarcinoma in this study include endoplasmic oxidoreductin-1-like protein B (*ERO1LB),* phosphodiesterase 1B, calmodulin-dependent *(PDE1B),* glutamate receptor, ionotrophic, AMPA 3 (*GRIA3*) and glutamate receptor, ionotropic, N-methyl-D-aspartate 3B (*GRIN3B*) (Figure 2) which were downregulated 5-fold, 8-fold and 4-fold, respectively. A complete list of genes downregulated in gastric cancer is provided as Supplementary Table 3.

### Bioinformatics analysis of differentially expressed genes

To gain functional insights from the genes that were differentially expressed in gastric cancer, we carried out gene set enrichment analysis and analysis of biological networks.

### Gene set enrichment analysis

GSEA was performed to determine differentially expressed genes that were enriched corresponding to specific functional pathways. Using GSEA, we searched a list of upregulated and downregulated genes in gastric cancer against 639 curated gene sets for canonical pathways from the molecular signature database (MsigDB) available at http://www.broad.mit.edu/gsea/msigdb/. Gene sets corresponding to ECM receptor interaction, focal adhesion and cell communication were found to be significantly upregulated while ribosome metabolism and calcium signaling pathways were significantly downregulated. As discussed above, some of the genes that were significantly downregulated including *GRIA3* and *GRIN3B* correspond to metabolic pathways downregualted in gastric cancer. As shown in Figure 2, closely related pathways share common genes and were coordinately regulated in gastric cancer. For example, ECM receptor interaction, focal adhesion, cell adhesion and cell communication share members of collagen family of genes (*COL11A2, COL1A2, COL1A1, COL3AI, COL4A1, COL5A3* and *COL5A2*) which were significantly upregulated in gastric cancer. Other genes that are common to these pathways and were upregulated include thrombospondins 1 and 2 (*THBS1* and THBS2) and *SPP1*.

### Biological network analysis

**Figure 3:**
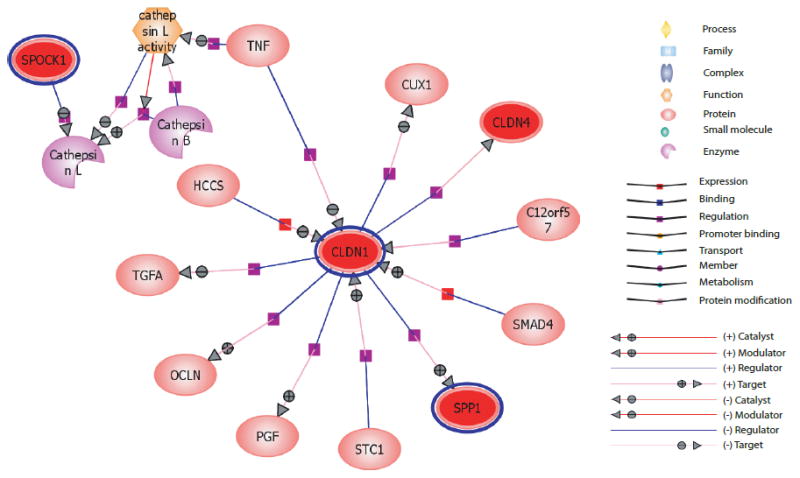
Biological network analysis of differentially expressed genes in gastric adenocarcinoma Illustration of sub-networks identified by network analysis. *SPP1, SPOCK1, CLDN1* and *CLDN4* overexpressed in gastric adenocarcinoma form a closely interconnected network through *CLDN1*. The key to the various processes/relationships are provided in the figure.

Biological network analysis was carried out using GeneSpring, which uses a natural language processing algorithm to generate an interaction database. Differentially expressed genes were given as input, which resulted in the generation of a complex network based on the connectivity between the genes. The generated network had various nodes which form highly interconnected sub networks. On overlaying expression values onto the network, four genes that were highly expressed in gastric adenocarcinoma (*SPP1*, *CLDN1*, *SPOCK1* and *CLDN4*), formed a distinguished subnetwork connected through *CLDN1* (Figure 3). Loss or rearrangements of tight junction proteins including claudins are implicated in epithelial to mesenchymal transition, which is a key event in metastasis and tumor progression [23]. Extracellular matrix proteins, which include proteoglycans, cell surface receptors, and cell adhesion molecules are known to play a crucial role in tumor progression [41]. Interaction of extracellular matrix proteins, *SPOCK1* and *SPP1*, the tight junction proteins *CLDN1* and *CLDN4* as shown in the network elucidates their possible role in gastric tumorigenesis. In addition, the function of less well characterized proteins such as *SPOCK1* could be elucidated by network analysis.

### Validation by immunohistochemical analysis

Among the molecules that were significantly upregulated in gastric cancer, we chose to validate two markers – villin-1, which has been previously reported in gastric cancer, and testican-1/ SPOCK1, which is a novel marker identified in this study. The above markers were chosen based on their biological significance, extent of upregulation and the availability of commercial antibodies.

### Immunohistochemical validation of known marker of gastric adenocarcinoma: Villin-1

**Figure 4:**
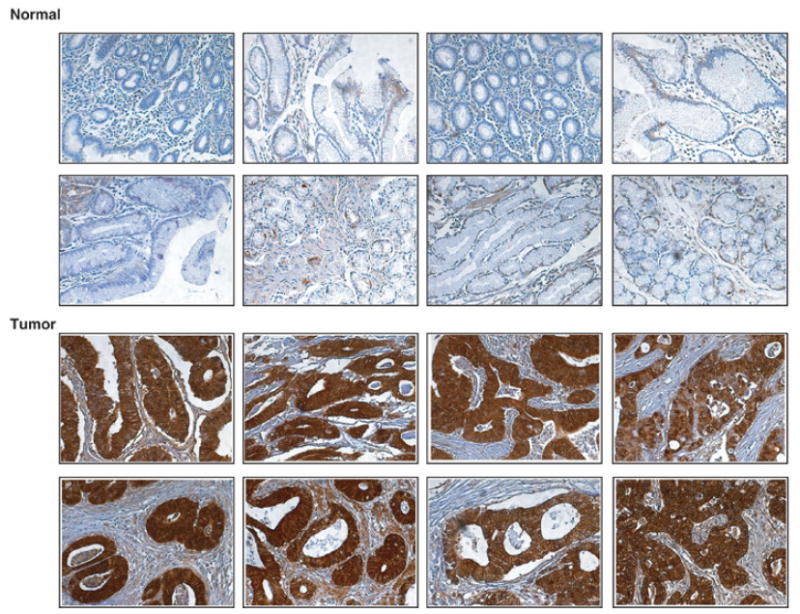
Immunohistochemical staining of Villin-1 in normal gastric tissue and gastric tumors Representative sections from tissue microarrays for normal gastric tissues and tumor tissues stained with anti-villin-1 antibody are shown.

Villin-1 (*VIL1*) is a calcium regulated actin binding protein of the intestinal brush border epithelium. It has been shown to be involved in epithelial mesenchymal transition [42]. In a recent study, it was reported to be a biomarker of cervical adenocarcinoma due to its preferential expression in adenocarcinoma but not in squamous cell carcinoma of the cervix [32]. Villin-1 has been used as a classical marker to represent the intestinal phenotype. It has been reported as a marker for intestinal type gastric adenocarcinoma [43]. It has also been shown to be expressed in gastric tubular adenocarcinomas [44] and to a lesser extent (5 out of 66 tested cases) in signet ring cell carcinomas [45]. In our study, villin-1 was found to be 9-fold upregulated at the mRNA level. By immunohistochemical analysis using tissue microarrays, we identified overexpression of villin-1 in 76% of the gastric adenocarcinoma tissues tested (217/282) as compared to normal epithelial tissues. Some of the cases stained for villin-1 are shown in Figure 4. As shown in the figure, villin-1 was predominantly localized to the cytoplasm.

### Immunohistochemical validation of novel marker of gastric adenocarcinoma: Testican-1/ SPOCK1

**Figure 5:**
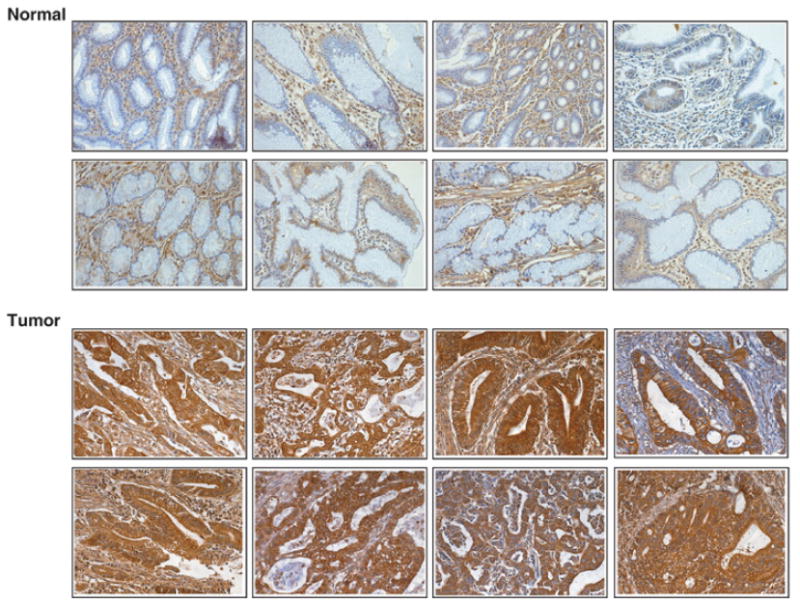
Immunohistochemical staining of Testican-1/SPOCK1 in normal and gastric tumors Representative sections from tissue microarrays for normal gastric mucosa and gastric adenocarcinoma stained with anti-testican-1 antibody are shown.

Testican-1/*SPOCK1* belongs to the family of calcium binding extracellular proteoglycans. Due to its modular architecture (SPARC/Osteonectin, CWCV and kazal like domains), it is referred to as SPOCK1. It was originally identified as a proteoglycan from seminal plasma which was later found to be expressed in other tissues [46,47]. It has been shown to be overexpressed in glioblastomas [48], prostate carcinomas [49] and gastrointestinal neuroendocrine carcinomas [50]. Due to its presence in body fluids such as blood and cerebrospinal fluid [47], it could be a suitable candidate as an early detection biomarker. In our study, we observed a 10-fold upregulation of *SPOCK1* in tumor tissues as compared to normal gastric mucosa. By immunohistochemical staining using tissue microarrays, we observed *SPOCK1* to be expressed in 80% (225/282) of the cases. Among them 56% (161/282) of the cases showed stronger expression of *SPOCK1* in tumor tissues as compared to normal tissues (Figure 5). As shown in the figure, *SPOCK1* showed cytoplasmic staining in all the cases tested. In addition to cytoplasmic staining, we could also observe staining in the stroma adjacent to both tumor and normal tissues.

## Conclusions

We have identified a number of candidates that were differentially expressed in gastric adenocarcinoma using whole human genome oligonucleotide arrays. We validated two candidates, testican-1 and villin-1, by immunohistochemical analysis using tissue microarrays on a larger panel of patients. Our study reveals that these markers could become clinically useful if tested further for their diagnostic, prognostic and therapeutic value. In addition, we have identified a number of novel candidates that are upregulated at mRNA level, which need further validation.

## Supplementary Material

S. Table-1

S. Table-2

S. Table-3

## Figures and Tables

**Table 1:** A partial list of upregulated genes previously reported in gastric adenocarcinoma.

	Gene Symbol	Protein	Citation	Fold-change
1	*CLDN1*	Claudin 1	Jung et al., 2010	23
2	*SPP1*	Secreted phosphoprotein 1	Imano et al., 2009	15
3	*KIAA1199*	KIAA1199	Matsuzaki et al., 2009	11
4	*TNFRSF11B*	Tumor necrosis factor receptor 11b	Ito et al., 2003	11
5	*THBS2*	Thrombospondin 2	Yang et al., 2007	10
6	*SERPINB5*	Serpin peptidase inhibitor, clade B, member 5	Song et al., 2007	10
7	*INHBA*	Inhibin, beta A	Zhang et al., 2010	10
8	*VIL1*	Villin-1	Osborn et al., 1988	9
9	*HMGA2*	High mobility group AT-hook 2	Motoyama et al., 2008	8
10	*SULF1*	Sulfatase 1	Junnila et al., 2010	8

**Table 2:** A partial list of novel genes upregulated in gastric adenocarcinoma.

	Gene Symbol	Protein	Features	Fold-change
1	*CLRN3*	Clarin 3	Transmembrane protein not well characterized	13
2	*SFRP4*	Secreted frizzled-related protein 4	Overexpressed in colorectal cancers	11
3	*SPOCK1*	Testican-1	Plasma proteoglycan with unknown function	10
4	*P4HA3*	Procollagen-proline, 2-oxoglutarate 4-dioxygenase	Enzyme not well studied	9
5	*TMEM158*	Transmembrane protein 158	Associated with colorectal carcinogenesis	9
6	*CHRDL2*	Chordin-like 2	Overexpressed in breast, lung and colon tumors	7
7	*GREM1*	Gremlin 1	Overexpressed in epithelial tumors	5
8	*ASPN*	Asporin	Novel marker identified in breast cancer	10
9	*ASH1L*	Ash1 (absent, small, or homeotic)-like	Reported as therapeutic target in lung cancer	7
10	*DOCK4*	Dedicator of cytokinesis 4	Known to play crucial role in cell migration	7
11	*HOXA11S*	Homeo box A11, antisense	Non coding RNA not well characterized	6
12	*PPAPDC1A*	Phosphatidic acid phosphatase type 2 domain containing 1A	Upregulated in breast cancer	6
13	*DPYSL3*	Dihydropyrimidinase-like 3	Phosphoprotein involved in neuroblastoma	5
14	*COMP*	Cartilage oligomeric matrix protein	Overexpressed in oral submucous fibrosis	6
15	*LPPR4*	Plasticity related gene 1	Membrane protein not well characterized	5
